# Network properties of human disease genes with pleiotropic effects

**DOI:** 10.1186/1752-0509-4-78

**Published:** 2010-06-04

**Authors:** Sreenivas Chavali, Fredrik Barrenas, Kartiek Kanduri, Mikael Benson

**Affiliations:** 1 The Unit for Clinical Systems Biology, University of Gothenburg, Medicinaregatan 5A, Gothenburg SE405 30, Sweden

## Abstract

**Background:**

The ability of a gene to cause a disease is known to be associated with the topological position of its protein product in the molecular interaction network. Pleiotropy, in human genetic diseases, refers to the ability of different mutations within the same gene to cause different pathological effects. Here, we hypothesized that the ability of human disease genes to cause pleiotropic effects would be associated with their network properties.

**Results:**

Shared genes, with pleiotropic effects, were more central than specific genes that were associated with one disease, in the protein interaction network. Furthermore, shared genes associated with phenotypically divergent diseases (phenodiv genes) were more central than those associated with phenotypically similar diseases. Shared genes had a higher number of disease gene interactors compared to specific genes, implying higher likelihood of finding a novel disease gene in their network neighborhood. Shared genes had a relatively restricted tissue co-expression with interactors, contrary to specific genes. This could be a function of shared genes leading to pleiotropy. Essential and phenodiv genes had comparable connectivities and hence we investigated for differences in network attributes conferring lethality and pleiotropy, respectively. Essential and phenodiv genes were found to be intra-modular and inter-modular hubs with the former being highly co-expressed with their interactors contrary to the latter. Essential genes were predominantly nuclear proteins with transcriptional regulation activities while phenodiv genes were cytoplasmic proteins involved in signal transduction.

**Conclusion:**

The properties of a disease gene in molecular interaction network determine its role in manifesting different and divergent diseases.

## Background

Decades-long research efforts have resulted in the identification of a large number of human disease genes [1-3]. Most of this research has been based on experimental and clinical studies of individual diseases and genes. A conceptually different approach was recently described, namely to study the network properties of human disease genes on a diseasome-wide scale. The studies were based on analyzing disease genes databases, such as the Online Mendelian Inheritance in Man (OMIM) [4]. The disease genes were classified as monogenic, polygenic or complex and their properties in molecular interaction networks were elucidated [5,6]. Further, it was shown that phenotypically similar diseases are often caused by functionally related genes [7-9]. This has led to the exploitation of molecular interaction networks to find novel candidate genes exploring neighbors of a disease-causing gene in a network as they are more likely to cause either the same or a similar disease [7,8].

Pleiotropy, in the context of human genetic diseases, implies that different pathological effects of different mutations within the same gene predispose an individual to different disorders [10]. While the previous studies have studied the properties of disease genes classified based on the number of genes involved in a phenotype, it is paramount to study the genes classified based on the number of phenotypes they are involved in. This would aid in identifying disease genes that are specific to diseases (specific genes), which can be exploited for therapeutic intervention. This would also help to find pleiotropic genes that are shared between different diseases (shared genes) to understand shared pathogenesis and hence mechanisms underlying co-morbidity [10-12]. Network properties of shared genes associated with phenotypically similar diseases have been examined so far, whereas those of pleiotropic genes with effects on divergent phenotypes and genes associated with specific diseases have not been examined. We hypothesized that the network properties of a gene in molecular interaction network and its tissue co-expression with its interactors determines the number of disease phenotypes it is associated with.

Here, we retrieved human disease genes and the associated diseases from Morbid Map (OMIM). We classified the shared disease genes into genes associated with phenotypically similar diseases (phenosim genes) and those that are associated with phenotypically divergent diseases (phenodiv genes) based on CIPHER score [13]. For instance, *AKT1 *which is associated with ovarian cancer, breast cancer, colorectal cancer and schizophrenia was classified as a phenodiv gene while *TYRP1 *which is associated with brown albinism and rufous albinism was classified as a phenosim gene. We demonstrated that shared genes were more central than specific genes while phenodiv genes were more central than phenosim genes. Shared genes had a higher number of disease gene interactors compared to specific genes. However, shared genes had a relatively restricted tissue co-expression with its interactors compared to specific genes. Essential genes, mutations in which lead to lethality, are known to be high degree nodes (hubs), thus occupying a central position in protein interaction network. When compared with specific, shared and phenosim genes essential genes had higher measures of centrality, as expected. However, essential genes and phenodiv genes had comparable connectivities (degrees) instigating us to explore for other network attributes of lethality and pleiotropy. We found that essential and phenodiv genes were intra-modular and inter-modular hubs, with the former being highly co-expressed with their interactors contrary to the phenodiv genes. Gene Ontology analysis identified the essential genes to be predominantly transcription factors residing in nucleus while phenodiv genes were cytoplasmic proteins involved in signal transduction. This study demonstrated that the effect of a disease gene on the number of different and phenotypically divergent diseases is associated with its properties in a molecular interaction network.

## Results

### Centrality of human disease genes in protein interaction network

**Figure 1:**
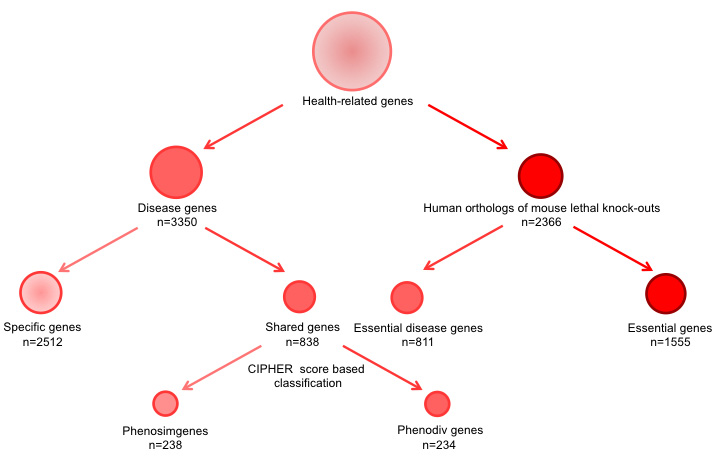
**Flow chart detailing how the different classes of genes were derived for this study**.

We retrieved a list of 3350 human disease genes from OMIM Morbid Map. If a gene is associated with only one disease it was classified as specific disease gene and if it is associated with more than one disease it belonged to the shared disease genes category (Figure 1). The importance of a node in a molecular network is often correlated to its centrality [14]. There are different measures that capture the centrality of a node in a network. We constructed a human protein interaction network using a modified version of CRG interactome [15] and investigated for differences in four different centrality measures namely degree, closeness, betweenness and eccentricity between specific and shared genes. The degree of a node provides the information about how many links (edges) that node has to other nodes in the network. Closeness is defined as the reciprocal average distance (number of links in the shortest path) to every other node- a node with high closeness is thus, on average, close in graph distance to the other nodes. Betweenness is a global centrality measure, which determines the centrality of a node in a network based on the total number of shortest paths going through the given node. Thus, nodes that occur on many shortest paths between other nodes have higher betweenness. The eccentricity of a node is the distance to the farthest reachable other node in a network, thus focusing on a maximal property where closeness focuses on an average.

**Figure 2:**
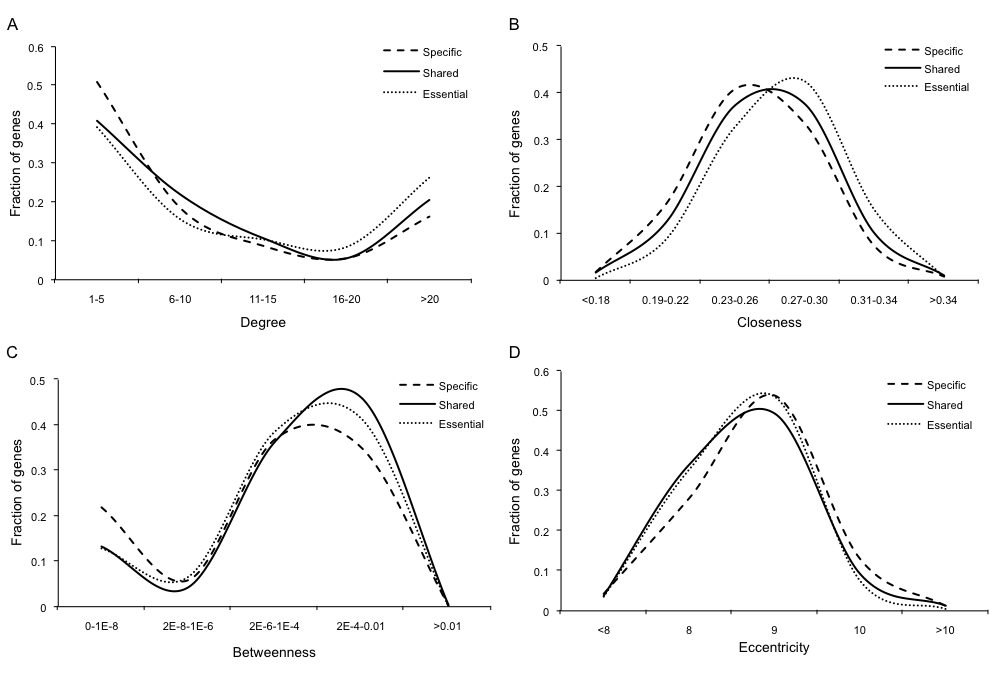
**Distribution profiles of measures of centrality A) Degree, B) Closeness, C) Betweenness and D) Eccentricity among the specific, shared and essential genes**. Shared genes show intermediate measures of centrality between essential genes and specific genes in human protein interaction network. Statistical comparisons of the measures of centrality between specific, shared and essential genes are presented in tables 1 and 2.

**Figure 3:**
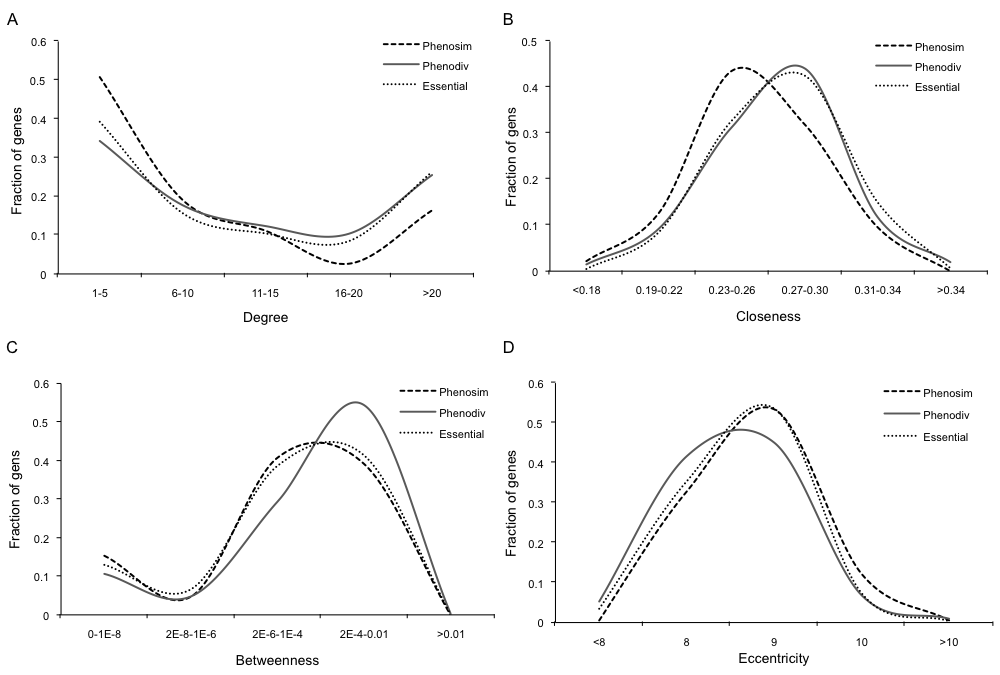
**Distribution profiles of measures of centrality A) Degree, B) Closeness, C) Betweenness and D) Eccentricity among the phenosim, phenodiv and essential genes**. Phenodiv genes have higher measures of centrality compared to Phenosim genes. Phenodiv genes have comparable measures of centrality to essential genes except higher betweenness. Statistical comparisons of the measures of centrality between phenosim, phenodiv and essential genes are presented in tables 1 and 2.

**Figure 4:**
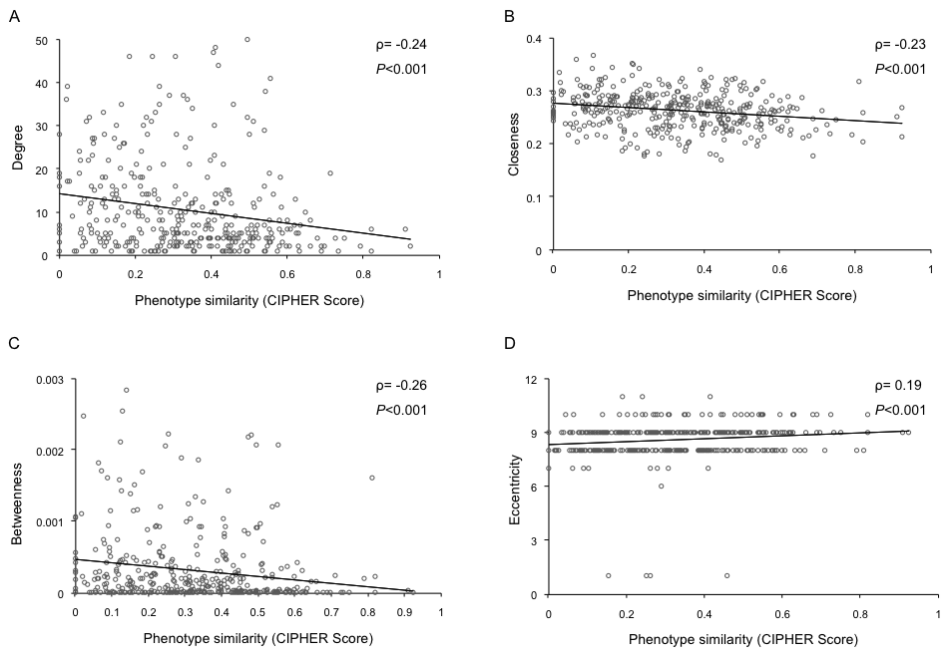
**Correlation of measures of centrality. A) Degree, B) Closeness, C) Betweenness, D) Eccentricity with phenotypic similarity estimated by CIPHER Score**. ρ represents Spearman's rho. The trend lines with negative slope in panels A, B and C indicate negative correlation of degree, closeness and betweenness with phenotypic similarity with the corresponding correlation coefficient and significance. The positive slope of the trend line in panel D demonstrates a positive correlation between eccentricity and phenotypic similarity. Thus, with the increasing phenotypic similarity, the respective disease associated genes show decreasing centrality. Very high degree values in the panel A have been removed in order to aid better visualization; however they have been considered for estimating correlation.

**Figure 5:**
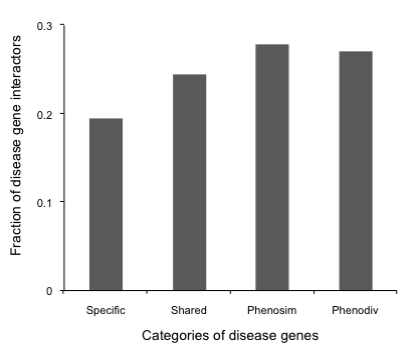
**Fraction of disease-gene interactors among those that interact with the four categories of disease genes**. Phenodiv and Phenosim genes have the highest number of disease-gene interactors while specific genes have the least.

Shared genes were more central than the specific genes in the protein interaction network as indicated by all the four measures of centrality (Table 1; Figure 2 panels A through D). These topological differences could be affected by shared genes in phenotypically similar diseases; such genes would be expected to be topologically similar to specific genes. To account for this we used CIPHER to distinguish between shared genes associated with phenotypically divergent and similar diseases (Phenodiv and phenosim genes respectively). Our analysis established that phenodiv genes were significantly more central than phenosim genes (Table 1; Figure 3 panels A through D). The observed differences in all the measures of centrality among the genes belonging to the four categories were well demonstrated by the distribution profiles (Figures 2 and 3). Further, phenotypic similarity of diseases (demonstrated by an increasing CIPHER score) showed a significant correlation with the centrality measures in the protein interaction network; we observed significant negative correlations of degree, closeness and betweenness with CIPHER score (Spearman's rho= -0.24, -0.23 and -0.26; *P *< 0.001 for all comparisons; Figure 4 panels A through C) while eccentricity was positively correlated (Spearman's rho= 0.19; *P *< 0.001; Figure 4D). In a protein interaction network human disease genes, relative to non-disease genes, are known to have a higher tendency to interact with protein products of other disease genes [4,5]. Since the shared genes are involved in many diseases they would be expected to interact with more disease genes compared to the specific ones. Confirming this, we observed that disease genes were overrepresented among the interactors of shared genes compared to those of specific ones (Figure 5; *P *< 0.001). This observation prompts us to speculate that there is a higher likelihood of finding a disease gene in the network neighborhood of shared genes. Phenodiv and phenosim genes had a comparable proportion of disease-gene interactors (*P *= 0.5). The enrichment of disease genes among the interactors of shared genes highlighted their role in shared pathogenesis.

**Table 1:** Comparison of measures of centrality of specific with shared genes and phenosim with phenodiv genes in human protein interaction network

Disease gene classes	Degree		Closeness		Betweenness		Eccentricity	
	*Mean ± S.D.*	P-value *	*Mean ± S.D.*	P-value *	*Mean ± S.D.*	P-value *	*Mean ± S.D.*	P-value *
Specific genes	13.29 ± 24.21	<0.001	0.26 ± 0.04	<0.01	3.3 × 10^-4 ^± 0.001	<0.001	8.65 ± 1.37	<0.001
Shared genes	16.28 ± 27.73		0.26 ± 0.03		5.9 × 10^-4 ^± 0.002		8.61 ± 1.06	
Phenosim genes	12.72 ± 20.72	<0.001	0.27 ± 0.04	<0.001	3.1 × 10^-4 ^± 0.0007	<0.001	8.75 ± 0.88	<0.01
Phenodiv genes	20.44 ± 34.85		0.27 ± 0.04		8.7 × 10^-4 ^± 0.003		8.47 ± 1.17	

*P-values were determined using Mann-Whitney U test

**Figure 6:**
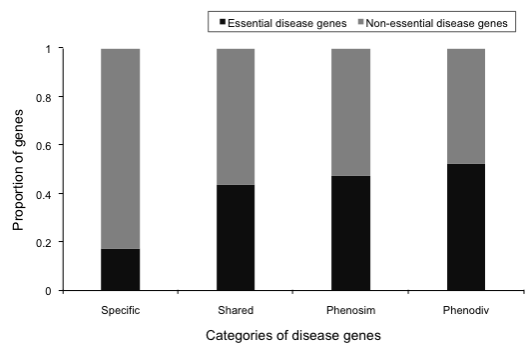
**Proportion of essential disease genes in the four categories of human disease genes**. Essential disease genes are defined as the orthologs of mouse genes that resulted in lethal phenotype upon knock-out, mutations in which lead to diseases in humans. Phenodiv genes have the highest number of essential disease genes while specific have the least.

Genes that are essential for early development, functional changes in which might lead to abortions, are termed as essential genes. Essential genes are known to show a tendency to be associated with hubs [4,14]. Some of the human orthologs of mouse lethal (essential) genes are known to be associated with human genetic diseases [4]. Essential genes associated with human diseases were classified as essential disease genes (n = 811) while others belonged to the essential genes set (n = 1555; Figure 1). As shared genes show higher centrality we speculated that these might be enriched with essential disease genes compared to specific genes. Shared genes showed an enrichment of essential disease genes compared to specific (44% to 17%; Fisher's Exact *P *< 0.001; Figure 6). Phenodiv and phenosim categories had comparable proportions of essential disease genes. These essential genes can be presumed to be vital for organism survival, sequence variants in which may lead to lethality. We observed that essential genes were significantly more central than specific and shared genes (Table 2; Figure 2). Phenosim genes differed significantly only in degree and closeness while phenodiv genes had all measures of centrality comparable to essential non-disease genes except betweenness. Phenodiv genes had significantly higher betweenness than essential genes (Table 2; Figure 3).

**Table 2:** Comparison of measures of centrality of Essential genes with that of specific, shared, phenosim and phenodiv genes in human protein interaction network

Measure	Essential	Specific	Shared	Phenosim	Phenodiv
	***Mean ± S.D.***	P-value*	P-value^*^	P-value^*^	P-value^*^
Degree	21.57 ± 35.89	<0.001	0.012	<0.001	0.44
Closeness	0.27 ± 0.03	<0.001	<0.001	<0.001	0.83
Betweenness	5.5 × 10^-4 ^± 0.002	<0.001	0.23	0.12	<0.001
Eccentricity	8.63 ± 0.87	<0.001	0.99	0.039	0.039

Mean ± S.D. values for the specific, shared, phenosim and phenodiv genes are provided in Table 1. *P-values were determined using Mann-Whitney U test

### Tissue-specificity of disease genes and their interactors

**Figure 7:**
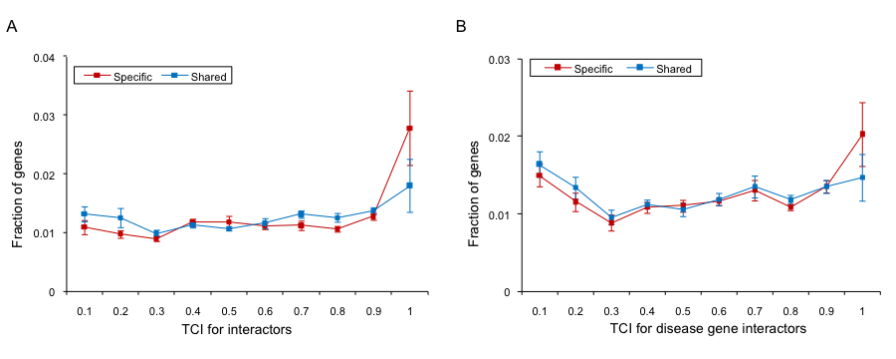
**Tissue co-expression of specific and shared genes with A) All interactors and B) Disease gene interactors**. The Tissue Co-expression Index (TCI) was calculated for a disease gene and its interactor as the fraction of the 79 tissues analyzed in which both were detected as expressed. Larger indices indicate that disease gene and the interactor are co-expressed in most tissues. The fractions of disease genes shown as the function of TCI indicate that specific genes are more co-expressed with their interactors as well as disease gene interactors compared to shared genes. The error bars represent SEM.

To discern whether centrality in a protein interaction network relates to a broader tissue distribution we checked for differences in the gene expression of the four categories of disease genes in 79 different human tissues [16]. We could not find any significant difference in the distribution of shared and phenodiv genes compared to specific and phenosim genes respectively (*P *= 0.05 and 0.23 respectively). A previous study suggests significant over-expression of disease genes and their complexes in normal tissues where defects cause pathology [17]. This led us to check for differences in the 'local' interactomes for the four classes of disease genes in the different tissues. For this, we integrated the protein interaction data with that of the gene expression information. When we checked for co-expression of interactors in the protein interaction network with those of the disease genes we identified that specific genes are more often co-expressed with their interactors than the shared genes (Figure 7A; Mann-Whitney *P *< 0.001; Mean ± S.D. = 45.9 ± 24.6 and 42.4 ± 24.1 respectively). We could not find any differences among phenodiv and phenosim genes (*P *= 0.45). Further, we identified that specific genes have a higher tendency of tissue co-expression with their disease-gene interactors compared to shared genes (Figure 7B; Mann-Whitney *P *< 0.001; Mean ± S.D. = 42.6 ± 24.6 and 40.2 ± 24.1 respectively). This, though somewhat unexpected, is in line with the earlier observation of increased co-expression of specific genes with their interactors compared to shared genes. Similarly, we could not find differences in co-expression of disease-gene interactors when shared genes were classified into phenodiv and phenosim genes (*P *= 0.05). Essential genes had higher tissue co-expression with their interactors compared to the different classes of disease genes except specific genes which had comparable proportions (Table 3). Notably, phenodiv genes had significantly lesser tissue co-expression.

**Table 3:** Comparison of tissue co-expression of interactors of Essential genes with that of specific, shared, phenosim and phenodiv genes

Gene Class	Tissue co-expression of interactors	
	*Mean ± S.D.*	P-value*
Essential	46.4 ± 23.2	
Specific	45.9 ± 24.6	0.42
Shared	42.4 ± 24.1	<0.001
Phenosim	42.5 ± 24.0	<0.001
Phenodiv	42.0 ± 22.9	<0.001

*P-values were determined using Mann-Whitney U test

### Pleiotropy and network modularity

**Figure 8:**
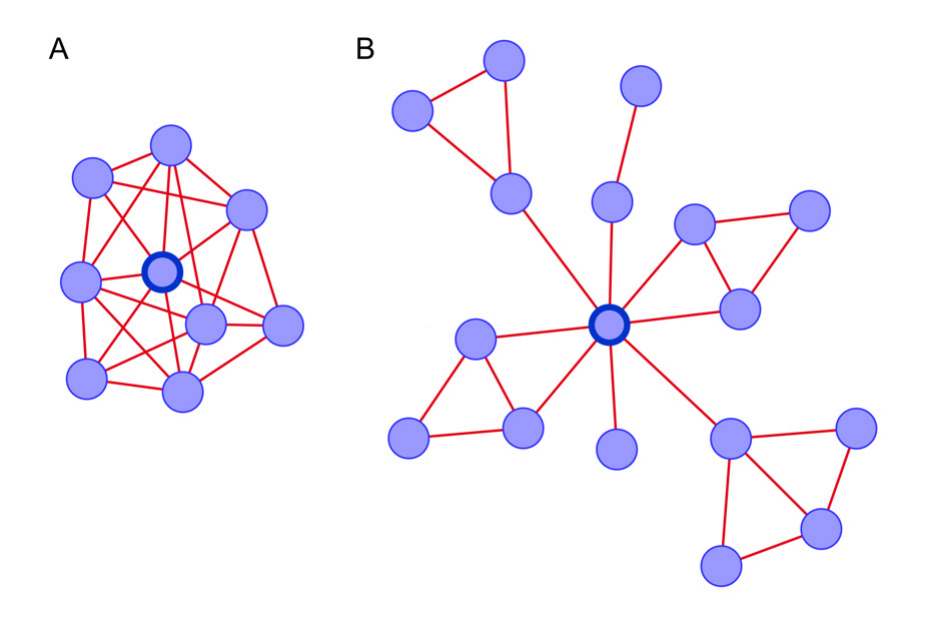
**A simplified illustration of the topological position of the protein product of A) Essential gene (Intra-modular hub) and B) phenodiv gene (Inter-modular hub) in the molecular interaction network**. The representative essential and phenodiv genes are marked in solid blue color node border. Both the classes of genes have same connectivities. Phenodiv genes have higher betweenness implying that these are proteins that occur on many shortest paths between other proteins in protein interaction network. On the other hand, essential genes have higher clustering coefficient suggesting the increased overall tendency of its interactors to form clusters.

In the human protein interaction network, phenodiv genes and essential genes had comparable degrees, closeness and eccentricity. However, phenodiv genes had greater global centrality as indicated by higher betweenness than essential genes (Mann-Whitney *P *< 0.001; Mean ± S.D. = 8.7 × 10^-4^ ± 0.003 and 5.5 × 10^-4^ ± 0.002 respectively). This prompted us to check for differences in clustering coefficient among essential and phenodiv genes. Clustering coefficient quantifies the cohesiveness of the neighborhood of a node and is defined as the ratio between the number of edges linking nodes adjacent to a node and the total possible number of edges among them. Thus, clustering coefficient characterizes the overall tendency of nodes to form clusters or groups. We observed that phenodiv genes had significantly lesser clustering coefficient than the essential genes (Mann-Whitney *P *= 0.018; Mean ± S.D. = 0.12 ± 0.19 and 0.17 ± 0.24 respectively). In addition, phenodiv genes showed relatively restricted tissue co-expression with their interactors in contrary to essential genes. Taken together, these results imply that these phenodiv and essential genes are classes of high degree genes (hubs) which are inter-modular and intra-modular respectively (Figure 8). Such hubs have been extensively studied recently [18] and biochemical differences have been reported between these two types of hubs. We hypothesized that the varying topological properties along with their different tissue co-expression profiles with their interactors could be explained by different biological functions. Hence, we used Gene Ontology (GO) to functionally characterize the essential and phenodiv genes for their cellular component, molecular function and biological process (Additional files 1 and 2). GO analysis of essential genes identified organelle, intracellular membrane bound organelle (1022 and 946 genes in contrast to a random expectation of 701.5 and 607.1 genes respectively; *P *< 0.001) to be the most significant cellular components with most of the genes in nucleus (737 genes as against a random expectation of 392.3 genes; *P *< 0.001). Contrarily, cytoplasm and membrane (123 and 121 genes in contrast with the random expectation of 82.9 and 88.1 respectively; *P *< 0.001) were overrepresented in phenodiv genes. The overrepresented molecular function categories for the essential genes include protein binding and nucleic acid binding (945 and 505 genes against random expectation of 588.1 and 280.8 respectively; *P *< 0.001) while apart from protein binding overrepresented molecular function classes for phenodiv genes were catalytic activity and signal transducer activity (84 and 62 genes to a random expectation of 65.2 and 28.2 respectively; *P *< 0.01 and <0.001 respectively). Intriguingly, both these classes of genes are involved in similar biological processes.

## Discussion

The phenotypic consequence of a variation in a gene is known to be affected to a large extent by the topological position of its protein product in the molecular interaction network. Thus, the functional importance of a gene is signified by its centrality in a protein interaction network. Previously, we and others have shown that the contribution of variations in a single gene to bring about an associated phenotype is a function of its centrality [4-6]. Accordingly, based on centrality different gene classes leading to a phenotype are ordered as essential genes (being the most central), monogenic disease genes, complex disease genes and non-disease genes (being the most peripheral). However, the network properties of a gene, mutations in which lead to various phenotypes have not been explored.

Based on the current understanding of the human protein interaction network and the results presented here, we demonstrated that the pleiotropic genes (shared genes) had an intermediate centrality compared with essential genes and genes associated with only one disease (specific genes). However, classification of the shared genes based on the similarity of the associated phenotypes demonstrated that phenodiv genes leading to divergent phenotypes were more central than phenosim genes. Thus based on increasing order of centrality these different disease genes could be arranged as Specific, Phenosim and Phenodiv genes. We note that the observed correlation of measures of centrality with phenotypic similarity provides support that the interpretations might not have been affected by considering median CIPHER value as a cut-off to classify phenosim and phenodiv genes.

**Figure 9:**
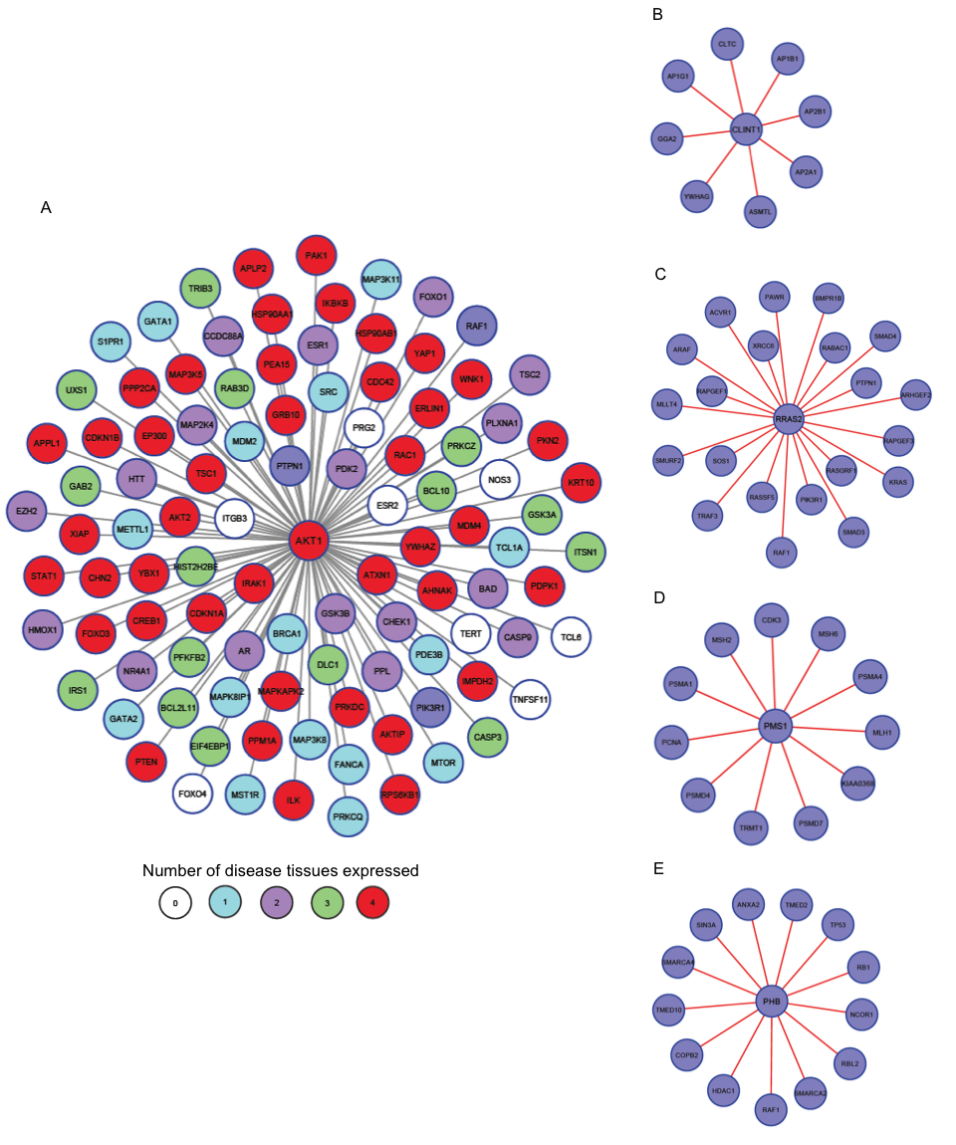
**Networks of interactors of disease genes in disease tissues**. A)	Network of interactors of the phenodiv gene *AKT1 *which is associated with schizophrenia, ovarian cancer, colorectal cancer and breast cancer. The color of the nodes indicates the number of disease tissues in which the interactors are expressed in. As indicated by the co-expression of interactors, AKT1 interacts with diverse interactors under different pathological conditions. AKT1 as a specific example of phenodiv genes demonstrates that phenodiv genes have more interactors (higher connectivity) and show relatively restricted co-expression with their interactors across different tissues. Network of interactors of specific genes- B) CLINT1 C) RRAS2 D) PMS1 and E) PHB associated with schizophrenia, ovarian cancer, colorectal cancer and breast cancer respectively. As has been observed for the class of specific genes, CLINT1, RRAS2, PMS1 and PHB have lesser number of interactors and are co-expressed with all their interactors in the respective disease tissue.

Co-expression with interactors is a prerequisite to bring about the function of a gene. Thus, specific genes with a very small network neighborhood would always be co-expressed with their interactors. Conversely, essential genes are hubs with high co-expression with their interactors. This attribute explains as to why mutations in these genes lead to lethality. Contrary to both specific and essential genes, shared genes showed decreased co-expression with their interactors. In addition to an intermediate centrality in the protein interaction network, this could be considered as an important functional property of genes with pleiotropic effects. For instance, the phenodiv gene *AKT1 *is associated with divergent phenotypes including schizophrenia, colorectal cancer, ovarian cancer and breast cancer. The network of its interactors varies among these diseases (Figure 9A) with some expressed in all disease tissues to very few not expressed in any. On the contrary, the disease-specific genes are co-expressed with all their interactors in the respective disease tissue. This is explicitly demonstrated by the co-expression of CLINT1 associated with schizophrenia with all its interactors in the brain region associated with the pathophysiology of the disease (Brodmann area 10: anterior prefrontal cortex; Figure 9B). Similarly, RRAS2, PMS1 and PHB which are associated only with ovarian cancer, colorectal cancer and breast cancer are co-expressed with their interactors in the respective disease tissues (Figure 9 panels C through E).

The similar measures of centrality between essential and phenodiv genes, except betweenness, led us to investigate the properties that determine essentiality (lethality of mutants) and pleiotropy. One of the most striking observations made here was that the essential genes and phenodiv genes were intra-modular and inter-modular hubs, with the former being highly co-expressed with its interactors contrary to the latter. Furthermore, essential genes were predominantly involved in transcription regulation while phenodiv genes in signal transduction.

This study could be affected by knowledge bias pertaining to the disease genes and their associated phenotypes as presented in OMIM, the human protein interaction network and the information on tissue expression. With an increasing number of genetic studies it is likely that some of the specific genes will be identified as shared and some of the phenosim as phenodiv genes. Based on the trend we observed here, it is tempting to speculate that essential disease genes in the specific and phenosim genes categories may have a higher likelihood for this transition. An expansion of knowledge of the diseases and disease genes, protein interactions and tissue expression would aid in better comprehension of the properties associated with genes causing pleiotropic effects. Further, it will be interesting to study the temporal co-expression of these genes with their interactors in various tissues.

## Conclusions

Here we demonstrated that the ability of a disease gene to influence the cellular network, signified by its centrality and tissue co-expression with its interactors, determines its pleiotropic effects.

## Methods

### Dataset

We obtained the list of human genetic diseases and associated 5024 diseases from the OMIM MorbidMap (downloaded in February 2009) [19]. Based on the number of disease associations the genes were then classified as specific- genes associated with one disease (n = 2512) and shared- those associated with more than one disease (n = 838). To determine the phenotypic divergence among diseases associated with the shared genes we used CIPHER (Correlating protein Interaction network and PHEnotype network to pRedict disease genes) [13]. CIPHER provides a similarity score of phenotypes for diseases in MorbidMap, based on their OMIM descriptions using Medical Subject Headings (MeSH) terms. Thus, a lower CIPHER score represents a higher phenotypic divergence. We retrieved CIPHER score for all diseases associated with the same gene and recorded the lowest score for each shared gene. This resulted in identification of 472 shared genes with phenotypic similarity/divergence information. Using the median CIPHER score (0.33) of the entire dataset as cut-off, we then categorized these genes into genes associated with phenotypically similar diseases (Phenosim genes with CIPHER scores ≥0.33; n = 238) and genes associated with phenotypically divergent diseases (Phenodiv genes with CIPHER scores < 0.33; n = 234). We defined essential genes (n = 2366) as previously described [4], by retrieving a list of human orthologs of mouse genes that resulted in lethal phenotype in embryonic and postnatal stages upon knockout as catalogued in Mouse Genome database [20]. Of the essential genes 811 were associated with human diseases and are classified as essential disease genes while 1555 essential non-disease genes were categorized as essential genes (Figure 1).

### Human protein interaction network

We constructed a human protein interaction network using a modified version of CRG interactome [15]. CRG interactome is by far the largest protein interaction network including protein-protein interactions supported by at least one direct experimental evidence demonstrating physical association between two human proteins. To validate proper annotation of the proteins, we retrieved Entrez gene identifiers for all the proteins that were listed in the CRG interactome with Ensembl gene identifiers. After removing entries that lacked Entrez gene identifiers, the modified CRG interactome consisted of 10,092 proteins with 79,211 interactions.

### Tissue expression

In order to examine differences in tissue distribution among the shared and specific genes, and the Phenosim and Phenodiv genes and co-expression with their interactors (from the human interaction network) we used previously described dataset of complete set of interactions with details of cells and tissues in which each interaction can occur [15]. This dataset was derived based on GNF Atlas expression data [16] which details tissue expression patterns of genes across 79 different non-disease human tissues. As previously described [15] we determined the expression of interactors for AKT1 in disease tissues- schizophrenia (GSE17612), colorectal cancer (GSE14333), breast cancer (GSE19615) and ovarian cancer (GSE18520). The datasets of disease tissues from patients were retrieved from NCBI Gene Expression Omnibus [21]. Expression of interactors of CLINT1, PMS1, PHB and RRAS2 were determined in schizophrenia, colorectal cancer, breast cancer and ovarian cancer disease tissues respectively. The interactors are considered co-expressed if they are expressed together in tissues in the datasets considered.

### Gene Ontology

Overrepresentation of Gene Ontology (GO) categories [22] was determined using the GOSim package from Bioconductor. Statistical significance estimation for overrepresented GO categories in real datasets (phenodiv and essential genes) was done by considering all GO categories without defining any levels in the GO hierarchy in order to avoid loss of information. We considered only categories with at least 5 total genes to prevent categories appearing to be significantly over-represented due to chance.

### Statistical Analysis

We compared the measures of centrality- degree, closeness, betweenness and eccentricity, between different classes of genes by Mann-Whitney U tests. Correlation between phenotypic similarity, as determined by CIPHER score and the measures of centrality was determined using Spearman's rank correlation. Different classes of disease genes were compared for disease essential genes using Fisher's Exact test. For GO analysis the number of genes in real dataset (N) with GO classifications was determined. In each GO category the number of genes in real dataset was counted. From the total number of genes represented in GOSim a set of N genes were randomly sampled and the number of genes present in each GO category was counted. This was repeated 10,000 to generate "randomly expected gene lists" for each GO category. Statistical significance was estimated as the ratio of number of times a GO category had more number of expected genes than observed for the real dataset genes to the number of random gene lists considered. Statistical analyses were performed using R.

## List of Abbreviations

OMIM: Online Mendelian Inheritance in Man; CIPHER: Correlating protein Interaction network and PHEnotype network to pRedict disease genes; GO: Gene Ontology; Phenosim genes: Genes associated with phenotypically similar diseases; Phenodiv genes: Genes associated with phenotypically divergent diseases;

## Authors' contributions

SC and FB designed, processed, interpreted the data and wrote the manuscript. KK has contributed in acquisition and interpretation of the data. MB has conceived and supervised the study. All the authors read and approved the final manuscript.

## Supplementary Material

Additional file 1 Over-represented Gene Ontology categories in Essential genes. Click here for file

Additional file 2 Over-represented Gene Ontology categories in phenodiv genes. Click here for file

## References

[B1] Jimenez-Sanchez G Childs B Valle D Human disease genes Nature 2001 409 853 855 10.1038/35057050 11237009

[B2] Peltonen L McKusick VA Genomics and medicine. Dissecting human disease in the postgenomic era Science 2001 291 1224 1229 10.1126/science.291.5507.1224 11233446

[B3] McKusick VA Mendelian Inheritance in Man and its online version, OMIM Am J Hum Genet 2007 80 588 604 10.1086/514346 17357067 PMC1852721

[B4] Goh KI Cusick ME Valle D Childs B Vidal M Barabási AL The human disease network Proc Natl Acad Sci USA 2007 104 8685 8690 10.1073/pnas.0701361104 17502601 PMC1885563

[B5] Feldman I Rzhetsky A Vitkup D Network properties of genes harboring inherited disease mutations Proc Natl Acad Sci USA 2008 105 4323 4328 10.1073/pnas.0701722105 18326631 PMC2393821

[B6] Barrenas F Chavali S Holme P Mobini R Benson M Network properties of complex human disease genes identified through genome-wide association studies PLoS One 2009 4 e8090 10.1371/journal.pone.0008090 19956617 PMC2779513

[B7] Aerts S Lambrechts D Maity S Van Loo P Coessens B De Smet F Tranchevent LC De Moor B Marynen P Hassan B Carmeliet P Moreau Y Gene prioritization through genomic data fusion Nat Biotechnol 2006 24 537 544 10.1038/nbt1203 16680138

[B8] Franke L van Bakel H Fokkens L de Jong ED Egmont-Petersen M Wijmenga C Reconstruction of a functional human gene network, with an application for prioritizing positional candidate genes Am J Hum Genet 2006 78 1011 1025 10.1086/504300 16685651 PMC1474084

[B9] Ideker T Sharan R Protein networks in disease Genome Res 2008 18 644 652 10.1101/gr.071852.107 18381899 PMC3863981

[B10] Park J Lee DS Christakis NA Barabási AL The impact of cellular networks on disease comorbidity Mol Syst Biol 2009 5 262 10.1038/msb.2009.16 19357641 PMC2683720

[B11] Zhernakova A van Diemen CC Wijmenga C Detecting shared pathogenesis from the shared genetics of immune-related diseases Nat Rev Genet 2009 10 43 55 10.1038/nrg2489 19092835

[B12] Lee DS Park J Kay KA Christakis NA Oltvai ZN Barabási AL The implications of human metabolic network topology for disease comorbidity Proc Natl Acad Sci USA 2008 105 9880 9885 10.1073/pnas.0802208105 18599447 PMC2481357

[B13] Wu X Jiang R Zhang MQ Li S Network-based global inference of human disease genes Mol Syst Biol 2008 4 189 10.1038/msb.2008.27 18463613 PMC2424293

[B14] Jeong H Mason SP Barabási AL Oltvai ZN Lethality and centrality in protein networks Nature 2001 411 41 42 10.1038/35075138 11333967

[B15] Bossi A Lehner B Tissue specificity and the human protein interaction network Mol Syst Biol 2009 5 260 10.1038/msb.2009.17 19357639 PMC2683721

[B16] Su AI Wiltshire T Batalov S Lapp H Ching KA Block D Zhang J Soden R Hayakawa M Kreiman G Cooke MP Walker JR Hogenesch JB A gene atlas of the mouse and human protein-encoding transcriptomes Proc Natl Acad Sci USA 2004 101 6062 6067 10.1073/pnas.0400782101 15075390 PMC395923

[B17] Lage K Hansen NT Karlberg EO Eklund AC Roque FS Donahoe PK Szallasi Z Jensen TS Brunak S A large-scale analysis of tissue-specific pathology and gene expression of human disease genes and complexes Proc Natl Acad Sci USA 2008 105 20870 20875 10.1073/pnas.0810772105 19104045 PMC2606902

[B18] Taylor IW Linding R Warde-Farley D Liu Y Pesquita C Faria D Bull S Pawson T Morris Q Wrana JL Dynamic modularity in protein interaction networks predicts breast cancer outcome Nat. Biotechnol 2009 27 199 204 10.1038/nbt.1522 19182785

[B19] Amberger J Bocchini CA Scott AF Hamosh A McKusick's Online Mendelian Inheritance in Man (OMIM) Nucleic Acids Res 2009 37 D793 D796 10.1093/nar/gkn665 18842627 PMC2686440

[B20] Blake JA Bult CJ Eppig JT Kadin JA Richardson JE Mouse Genome Database Group The Mouse Genome Database genotypes::phenotypes Nucleic Acids Res 2009 37 D712 D719 10.1093/nar/gkn886 18981050 PMC2686566

[B21] Barrett T Troup DB Wilhite SE Ledoux P Rudnev D Evangelista C Kim IF Soboleva A Tomashevsky M Marshall KA Phillippy KH Sherman PM Muertter RN Edgar R NCBI GEO: archive for high-throughput functional genomic data Nucleic Acids Res 2009 37 D885 90 10.1093/nar/gkn764 18940857 PMC2686538

[B22] Ashburner M Ball CA Blake JA Botstein D Butler H Cherry JM Davis AP Dolinski K Dwight SS Eppig JT Harris MA Hill DP Issel-Tarver L Kasarskis A Lewis S Matese JC Richardson JE Ringwald M Rubin GM Sherlock G Gene ontology: tool for the unification of biology. The Gene Ontology Consortium Nat Genet 2000 25 25 29 10.1038/75556 10802651 PMC3037419

