# Supplementary material for: Network properties of human disease genes with pleiotropic effects
Source: BMC Syst Biol. 2010 Jun 4;4:78. doi: 10.1186/1752-0509-4-78 (PMC2892460; doi:10.1186/1752-0509-4-78)
Supplement: Additional file 1 — Over-represented Gene Ontology categories in Essential genes. [file 1752-0509-4-78-S1.PDF]

Additional File 1: Over-represented Gene Ontology categories in Essential genes

Go category	Go Term	Essential genes	Expected genes	*P*-value
**Cellular Component**				
GO:0005623	cell	1408	1238.3	<0.001
GO:0044464	cell part	1408	1238.2	<0.001
GO:0005622	intracellular	1189	888.5	<0.001
GO:0044424	intracellular part	1170	845.3	<0.001
GO:0043226	organelle	1022	701.5	<0.001
GO:0043229	intracellular organelle	1022	701.2	<0.001
GO:0043227	membrane-bound organelle	946	607.3	<0.001
GO:0043231	intracellular membrane-bound organelle	946	607.1	<0.001
GO:0005634	nucleus	737	392.3	<0.001
GO:0005737	cytoplasm	673	547.3	<0.001
**Molecular Function**				
GO:0005488	binding	1340	1029.1	<0.001
GO:0005515	protein binding	945	588.1	<0.001
GO:0003676	nucleic acid binding	505	280.8	<0.001
GO:0003824	catalytic activity	473	429.8	<0.01
GO:0003677	DNA binding	453	195.2	<0.001
GO:0043167	ion binding	404	355.1	<0.001
GO:0046872	metal ion binding	400	347.9	<0.001
GO:0030528	transcription regulator activity	388	117.9	<0.001
GO:0043169	cation binding	366	322.9	<0.01
GO:0003700	transcription factor activity	284	79.4	<0.001

Go category	Go Term	Essential genes	Expected genes	*P*-value
**Biological Process**				
GO:0009987	cellular process	1358	1014.4	<0.001
GO:0065007	biological regulation	1011	593.7	<0.001
GO:0050789	regulation of biological process	965	563.0	<0.001
GO:0008152	metabolic process	963	673.5	<0.001
GO:0050794	regulation of cellular process	948	548.12	<0.001
GO:0044237	cellular metabolic process	928	620.1	<0.001
GO:0044238	primary metabolic process	925	606.5	<0.001
GO:0043170	macromolecule metabolic process	848	520.8	<0.001
GO:0043283	biopolymer metabolic process	765	419.0	<0.001
GO:0032502	developmental process	658	252.2	<0.001
