# Supplementary material for: Network properties of human disease genes with pleiotropic effects
Source: BMC Syst Biol. 2010 Jun 4;4:78. doi: 10.1186/1752-0509-4-78 (PMC2892460; doi:10.1186/1752-0509-4-78)
Supplement: Additional file 2 — Over-represented Gene Ontology categories in phenodiv genes. [file 1752-0509-4-78-S2.PDF]

Additional File 2: Over-represented Gene Ontology categories in phenodiv genes

Go category	Go Term	Phenodiv genes	Expected genes	*P*-value
**Cellular Component**				
GO:0005623	cell	218	187.6	<0.001
GO:0044464	cell part	218	187.6	<0.001
GO:0005622	intracellular	165	134.5	<0.001
GO:0044424	intracellular part	164	127.9	<0.001
GO:0043226	organelle	131	106.1	<0.001
GO:0043229	intracellular organelle	130	106.1	<0.01
GO:0005737	cytoplasm	123	82.9	<0.001
GO:0016020	membrane	121	88.1	<0.001
GO:0043227	Membrane-bound organelle	116	91.9	<0.001
GO:0043231	intracellular membrane-bound organelle	116	91.8	<0.001
**Molecular Function**				
GO:0005488	binding	207	155.9	<0.001
GO:0005515	protein binding	156	89.0	<0.001
GO:0003824	catalytic activity	84	65.2	<0.01
GO:0004871	signal transducer activity	62	28.2	<0.001
GO:0060089	molecular transducer activity	62	28.2	<0.001
GO:0000166	nucleotide binding	52	27.1	<0.001
GO:0004872	receptor activity	49	22.8	<0.001
GO:0017076	purine nucleotide binding	49	23.4	<0.001
GO:0032553	ribonucleotide binding	46	22.5	<0.001

Go category	Go Term	Phenodiv genes	Expected genes	*P*-value
GO:0032555	purine ribonucleotide binding	46	22.5	<0.001
**Biological Process**				
GO:0009987	cellular process	200	153.6	<0.001
GO:0065007	biological regulation	161	89.9	<0.001
GO:0032501	multicellular organismal process	151	43.2	<0.001
GO:0050789	regulation of biological process	148	85.3	<0.001
GO:0050794	regulation of cellular process	143	83.0	<0.001
GO:0008152	metabolic process	140	102.1	<0.001
GO:0032502	developmental process	135	38.2	<0.001
GO:0044237	cellular metabolic process	132	94.0	<0.001
GO:0044238	primary metabolic process	124	92.0	<0.001
GO:0007275	multicellular organismal development	115	27.6	<0.001
